# Myocarditis Mimicking an Acute Coronary Syndrome: A Case Related to *Salmonella enteritis*

**DOI:** 10.1155/2009/931853

**Published:** 2009-11-22

**Authors:** B. Rossetti, G. Nguisseu, A. Buracci, L. Migliorini, G. Zanelli

**Affiliations:** Department of Molecular Biology, Infectious Diseases Clinic, Siena University, 53100 Siena, Italy

## Abstract

Infective myocarditis is most commonly due to a viral infection; occasionally it has been related to bacteria. Gastrointestinal infections associated with myocarditis have only rarely been described in young people, and the pathogenesis is unclear. We report a case of myocarditis mimicking an acute coronary syndrome (ACS) in a patient hospitalized for fever and diarrhoea. *Salmonella enteritidis* was isolated from stool, and no other pathogens were found. The coronary angiography was normal, and there were not other coronary artery risk factors, other than hypertension. The patient was treated with ciprofloxacin, acetylsalicylate acid, and ramipril with rapid clinical improvement and normalization of cardiac abnormalities. Final diagnosis of *Salmonella enteritis* and related myocarditis was made based on clinical, laboratory, ECG and echocardiographical findings.

## 1. Introduction

 A large variety of infections, systemic diseases, drugs, and toxins have been associated with the development of myocarditis. The clinical manifestation is often oligosymptomatic, so the real incidence could be underestimated [1]. Infective myocarditis is most commonly due to a viral infection; occasionally it has been related to bacteria [1, 2]. Myocarditis secondary to gastrointestinal infections has only rarely been described in young people, and the pathogenesis is uncertain [2]. We report a case of myocarditis mimicking an acute coronary syndrome in a patient with Salmonella enteritis. 

## 2. Case Presentation

 A 36-year-old Cameroonian man was admitted to the hospital with fever, abdominal pain, and watery diarrhoea (not bloody). Two days before the onset of the symptoms he had attended a wedding, where he ate a buffet meal. He suffered from asthma and hypothyroidism. Other than hypertension, he did not have other coronary artery disease risk factors.

On examination, he was suffering from painful abdomen; temperature was 38.8°C, blood pressure 160/90, heart rate 96 beats per minute, and respiratory rate 20 per minute. Cardiac examination was normal; respiratory examination revealed bronchostenosis. Laboratory investigations showed normal blood counts and serum electrolytes; C reactive protein was 0.64 mg/dl (reference range <0.35 mg/dl). Chest X-ray and abdominal ultrasound were normal. The patient was initiated on Ciproxin (400 mg bid i.v.) when *Salmonella enteritidis* group B was isolated from stool. Blood culture was sterile, and the remaining bacteriological and viral examination of throat was negative. Shigella, Campylobacter, Yersinia, C. difficile toxin, Adenovirus and Rotavirus were not detected from the stool. On the third day of hospitalization the patient experienced severe retrosternal chest pain, radiating to the left arm and the interscapular area. Creatine kinase (CK) and CK-MB were elevated to 662 UI/L (reference range <170 UI/L) and 54 UI/L (reference range <10% of CK). Troponin T was 0.59 ng/mL (reference range <0.01 ng/mL). The electrocardiogram (ECG) showed ST-wave elevations in D1, AVL, V2, and V3. The echocardiography (ECHO) revealed hypokinetic areas in the middle basal, anterior-basal, lateral walls, with left ventricle ejection fraction 57%, concentric hypertrophy, without pericardial effusion. An acute coronary syndrome was suspected, and the patient was admitted to the coronary care unit for intensive cardiac monitoring. The coronary angiography showed the absence of coronary disease or spasm. It was treated with acetylsalicylate acid (ASA), enoxaparin sodium, ramipril, and atorvastatin. Over the course of 24 hours the chest pain resolved, and the controlled echo showed normal left ventricle function without signs of myocardial infarction. The patient remained hemodynamically stable and cardiac enzymes normalized on the third day. The patient was discharged from the hospital after 15 days of treatment. At home he continued ASA and ramipril; after three months he was well. 

Final diagnosis of Salmonella enteritis and related myocarditis was made based on clinical, laboratory, ECG, and echocardiographical findings. Enterovirus was not detected from throat swab. 

## 3. Discussion

 Myocarditis is an insidious disease, and its real incidence remains underestimated [1]. Although a large variety of causes have been associated with the development of myocarditis, the etiology often is unknown. Infective myocarditis is most commonly due to a viral infection, overall Enterovirus; occasionally it has been associated to bacterial infections [1]. Cardiac complications are rarely described in association with salmonellosis [3–5], shigellosis [6], and *Campylobacter jejuni* [7–9]. Generally the patients are young people without coronary artery disease risk factors. The pathophysiology is not well understood, and several theories have been developed. The potential mechanism includes direct invasion of myocardium, influence of toxins, and immunologically mediated myocardial damage [1]. In our patient the negative blood culture and the chest pain developed 3 days after the onset of diarrhoea, similarly to other cases, may indicate a cardiac toxic damage. Molecular characterization of the isolated strain could be of interest. Myocardial inflammation is usually oligosymptomatic but it can progress to a chronic cardiomyopathy and it is considered a leading cause of sudden death in patient under 40 years old [1].

The diagnosis of myocarditis made based on clinical, laboratory, ECG, and echo findings is not always easy. In our case, clinical manifestations simulated an acute coronary syndrome; the coronary angiography has been necessary to reach the diagnosis. In the case described, the absence of coronary disease or spasm, the temporal coincidence with gastrointestinal complaints, and the full recovery after antibiotic therapy allowed us to make a diagnosis of myocarditis caused by *Salmonella enteritidis*. The proper diagnosis of myocarditis requires an endomyocardial biopsy; although this invasive examination is recommended in few circumstances and often offers postmortem information; recently cardiac magnetic resonance (CMR) imaging has been demonstrated to be useful for the diagnosis [10]. The prognosis of myocarditis is not always benign; however the absence of left ventricular dilated and the full return of ventricular function (presented by our patient) seem associated with a low risk of progression to dilatated cardiomyopathy [9]. No established treatment regimen exists, and it relates to the patient hemodynamic status [9]. 

## 4. Conclusions

 Myocarditis can be a severe complication of infectious disease. This paper describes a rare case of cardiac involvement as a complication of *Salmonella enteritidis* infection and shows the importance of cardiac monitoring in patients with bacterial enteritis, even if they have not coronary artery disease risk factors.
